# The Relationships between Parenting Practices and Child Health-Related Behaviors in Children with Intellectual Disability: The Moderating Role of Child Body Weight Status

**DOI:** 10.3390/nu14245206

**Published:** 2022-12-07

**Authors:** Yan Sun, Rashmi Supriya, Yang Gao, Siyue Yu, Aiwei Wang, Xiaoting Ou, Dan Tao, Julien S. Baker

**Affiliations:** 1 Department of Sport, Physical Education and Health, Hong Kong Baptist University, Hong Kong 999077, China; 2 Centre for Health and Exercise Science Research, Hong Kong Baptist University, Hong Kong 999077, China; 3 JC School of Public Health and Primary Care, The Chinese University of Hong Kong, Hong Kong 999077, China; 4 College of Physical Education, Yangzhou University, Yangzhou 225012, China; 5 Department of Government and International Studies, Hong Kong Baptist University, Hong Kong 999077, China

**Keywords:** children, intellectual disability, parenting practices, health-related behaviors, eating, body weight status

## Abstract

This study aimed to examine the associations between parenting practices and child health-related behaviors, and the moderating role of child body weight status in children with intellectual disability (ID). A cross-sectional study was conducted among a sample of children with ID in Hong Kong; 440 participants were included in this study. All the variables investigated were collected from questionnaires, except body weight status, which was objectively measured. Logistic regression analysis was used to examine the associations between parenting practices and children’s unhealthy behaviors. Interaction items were added to investigate the moderation effect of child body weight status, adjusting for significant background characteristics. Results showed that the parenting practices of “restricting access to unhealthy food and sedentary behaviors (RA)” (OR range: 0.63–0.64) and “using food or sedentary behaviors as rewards (UR)” (OR range: 1.28–1.60) were significantly associated with some eating behaviors, but not with sedentary behaviors. Body weight status significantly moderated these associations. Only RA showed favorable effects on some eating behaviors in children with overweight and obesity (OR range: 0.17–0.28), whereas the effects of UR differed by body weight status. Future research should focus on developing educational interventions which encourage parents to use practices that are tailored towards children’s individual characteristics.

## 1. Introduction

Overweight and obese status in children and adolescents is an imminent crisis in public health and prevalence rates continue to rise. Previous research has established that children with intellectual disability (ID) are more vulnerable to overweight and obese status and developing unhealthy behaviors. Children with ID may face more developmental problems in processing information (e.g., cognitive impairment, communication disorders, limited mental functioning), and consequently they have difficulties in understanding and learning health knowledge and developing healthy behaviors [1,2,3,4]. In Hong Kong, there were 7700 children with ID studying in special schools in 2014 [5]. Our previous study reported that the combined prevalence of overweight and obese status in children with ID was 31.3% (vs. non-ID counterparts: 18.7–19.9%), and was associated with unhealthy behaviors (i.e., longer durations of sedentary behavior, higher consumption of sweetened drinks and of fried food) [6]. These unhealthy behavioral patterns develop during childhood and tend to persist into adulthood, which further results in greater risks of serious health issues (e.g., adult obesity, cardiovascular disease) [7,8]. Substantial evidence suggests that actions and attitudes from parents can have a significant impact on children’s health-related behaviors in childhood. These actions and attitudes can support children in developing healthy habits or can use specific parenting practices to control the availability and accessibility of healthy eating and exercise opportunities [9]. For children with ID, they may need more help to achieve healthy behaviors, and thus parents have significant roles in parenting a child with ID [10,11].

Parental practices, referring to parental actions or behaviors, are more content-specific to the domain of children’s eating and exercise compared with general parenting styles, which focus on the values, beliefs, and the emotional climate that parents convey to children as they develop. In addition, parenting practices may be more changeable and promising for establishing healthy habits in childhood [12,13]. Many studies have investigated the associations between parenting practices and child health-related behaviors; however, these produced conflicting results. For example, some experimental and longitudinal studies suggest that restricting access to unhealthy food was associated with a higher consumption of unhealthy food (e.g., sweetened drinks, snacks) and lower intakes of fruits and vegetables [14,15,16,17]. Contradictory findings were noted from cross-sectional studies, demonstrating that food restriction had favorable effects on children’s eating behaviors [18,19,20,21]. Moreover, other parenting practices (e.g., pressure to eat, monitoring) regarding eating were reported to be associated with both desirable (e.g., [20,22,23]) and undesirable (e.g., [24,25]) eating behaviors. In addition, parenting practices regarding physical activity (PA) and sedentary behaviors presented mixed findings. Restricting and monitoring children’s amounts of screen watching were found to be associated with less sedentary behavior and increased PA [23,26,27,28], whereas some findings did not support these associations [29]. In addition, a variation in parenting practices measures across studies may be another contributory factor to inconsistent findings [14]. In the current study, parenting practices regarding children’s eating and exercise were measured in five domains: monitoring food intake and PA; restricting access to unhealthy food and sedentary behaviors; pressure to eat; reinforcement regarding eating and PA; and using food or sedentary behaviors as rewards. 

The solutions to interpret conflicting findings on associations between parenting practices and child health-related behaviors may lie in the interactions between certain child aspects and parenting practices. Some child characteristics (e.g., body weight status) may moderate these associations [30]. For instance, a previous study identified the moderation effect of child body weight status on associations between restriction and dietary behaviors, presenting favorable effects for eating in children with normal weights, but not in children with overweight status [31]. A further study found that the effects of parenting practices on child health-related behaviors were not affected by child body weight status [32]. Evidence of the moderation effect of child body weight on these associations is still lacking. Children with ID are more vulnerable to unhealthy behaviors, overweight and obese status, and parenting practices seem more significant in establishing healthy habits compared with typically developing peers [33]. However, no research clarifies the moderating role of child body weight status of these associations in children with ID.

We previously investigated the associations between parenting practices and the risk of developing overweight and obese status in children with ID [6], whereas the effects of parenting practices on child health-related behaviors and the moderating role of child body weight status are unknown. Based on previous findings and evidence, we developed this study to firstly examine the associations between parenting practices and child health-related behaviors, and secondly, to examine whether these associations would be moderated by child body weight status in children with ID.

## 2. Methods

### 2.1. Study Design and Participants

We conducted a cross-sectional study among students in special schools for children with mild (intelligence quotient: 50–69) and moderate (intelligence quotient: 35–49) ID in Hong Kong between June and November 2015 [34]. Sample size was estimated using a sample size formula for comparing proportions between a sample and the population [35,36]. Using a significance level of 5% and a power level of 80%, a sample size of 397 was estimated. Invitation letters were sent to all schools (*n* = 31), of which 12 schools consented to participate. We then invited all eligible students in the 12 schools who provided written informed consent forms signed by parents or guardians to participate in this study. The Research Ethics Committee of Hong Kong Baptist University ethically approved the study (Ref. No.: FRG1/13-14/067). A detailed description of the study methodology has been reported elsewhere [6]. Participants who met the following criteria participated in this study: (1) non-boarding students (i.e., living with parents); (2) respondents were parents; (3) valid data of parenting practices, child body weight status and health-related behaviors. 

### 2.2. Measures

The participants’ background characteristics, health-related behaviors (including PA, sedentary behaviors, and eating habits) and related parenting practices were reported by their parents/guardians together using a self-administered questionnaire. Children’s body weight status was determined from a calibrated weighing scale and height from a stadiometer [6]. The questionnaire used was reliable for the study population, with an average Kappa of 0.71 obtained in a test–retest evaluation.

#### 2.2.1. Background Characteristics of the Participants

The participants’ gender, age, ID level, and comorbidities (including Autism, attention deficit hyperactivity disorder (ADHD), Down syndrome, and epilepsy) were collected using the self-administered questionnaire. In addition, their parents’ education, occupation, marital status, reported height and weight, as well as the respondent parents’ age, were also obtained. Parental obesity was defined using a body mass index (BMI) cutoff point of 25 kg/m^2^ for Asian adults, where BMI was calculated using the self-reported height and weight [37].

#### 2.2.2. Parenting Practices

Parenting practices on children’s PA, sedentary behaviors and eating were collected using a Chinese version of a 17-item 5-point Likert-type scale [38]. It consists of the following five subscales: diet and PA monitoring (MO, six items; example question: “How often do you keep track of sweetened snacks (e.g., candy, ice cream, cake) that your child eats?”); restricting access to unhealthy food and sedentary behaviors (RA, four items; example question: “On weekdays, I limit the amount of TV or videos my child is watching.”); pressure to eat more (PE, three items; example question: “My child eats less than what he/she should eat if I do not intervene.”); reinforcement regarding children’s eating and PA (RF, two items; example question: “I will praise my child if he/she actively increases PA.”); and using food or sedentary behaviors as rewards (UR, two items; example question: “I would offer TV, video, or video games to my child as a reward for good behaviors.”). The scale has sound validity and reliability in both Chinese and Caucasian populations [23,38]. Supplementary Table S1 lists all items and ratings for the scale. The average score of each subscale (ranged from 1 to 5) was calculated for each participant, with a higher score indicating more frequent practices.

#### 2.2.3. Children’s Health-Related Behaviors

Health-related behaviors included PA, sedentary behaviors, and eating habits. Children’s PA and sedentary behaviors in a typical week were subjectively measured with the Chinese version of the Global Physical Activity Questionnaire (GPAQ) [39], modified for school children. For example, the context of performing PA at “work” was replaced by “school”, and fitted examples, respectively. Then, daily minutes of moderate- to vigorous-intensity PA (MVPA) and those of sedentary behaviors were calculated. Insufficient PA was defined with MVPA < 60 min/day [40], while more sedentary behaviors were determined with 4 h/day as a cutoff point. Eating habits in a typical week were reported on a frequency of food consumption questionnaire, which was developed and validated for children in Hong Kong [41]. Lower fruit consumption (<2 servings/day), lower vegetable consumption (<3 servings/day), higher fried food consumption (≥once/day), higher sweetened drink consumption (≥once/day), higher snack consumption (≥twice/day) and breakfast skipping (≤6 times/week) were then defined.

#### 2.2.4. Child Body Weight Status

Well-trained investigators measured children’s height (in cm) and weight (in kg) at school in the morning following a standard protocol. Overweight and obese status was defined based on international age- and gender-specific BMI cutoff points recommended by Cole et al. [42].

### 2.3. Data Analysis

Categorical variables were described with counts and percentages, while continuous variables were presented with means and standard deviations (SDs). Independent sample t-tests and chi-square tests were employed to compare: (1) differences in all study variables between the original sample of all participants and the selected sub-sample of those fulfilling the selection criteria for this paper; (2) differences in parenting practices and health-related behaviors between children with overweight and obese status and children with non-overweight and obese status in this sub-sample. Univariate logistic regression was performed to examine relationships between children’s background characteristics and their behaviors. Multivariate logistic regression was fitted to examine the moderating role of child body weight status on parenting practices and unhealthy behaviors, adjusting for children’s age, gender, and the background characteristics with *p* < 0.10 used in the univariate analysis. Main effects along with the adjusted variables were forcedly entered in Block 1; interaction items between child body weight status and parenting practices were forward selected in Block 2, with *p* = 0.10 and *p* = 0.15 as entry and removal criteria, respectively [43]. The moderating effect of child body weight status was then identified if an interaction item reached significance (*p* < 0.05). Finally, those multivariate regression models with significant interaction items were further performed after stratification by child body weight status with the same adjusted variables as the previous step. Crude odds ratios (CORs) and adjusted odds ratios (AORs), with 95% confidence intervals (95% CIs), were derived from the univariate analysis and multivariate analysis, respectively. The SPSS Statistics version 26.0 was used to conduct all analyses and statistical significance was defined as *p* < 0.05.

## 3. Results

**Figure 1:**
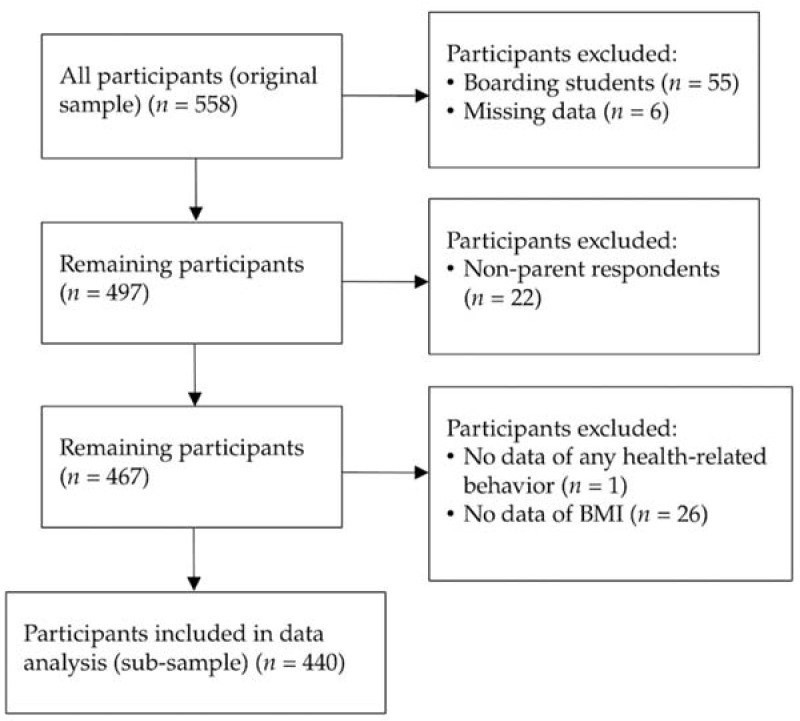
Flow chart of participant selection.

There were 558 participants in the original sample, of which 118 did not fulfill the selection criteria and were therefore excluded. The remaining 440 participants in the sub-sample were included in data analysis (Figure 1). There was no significant difference in any study variable between the original sample (*n* = 558) and the sub-sample (*n* = 440).

Among the participants in the sub-sample, around two-thirds were male (69.8%); over half (54.8%) were aged from 6 to 12 years; 31.6% were overweight and obese. Table 1 lists all the background characteristics of the participants. 

Table 2 compared mean scores of parenting practices and the prevalence of unhealthy behaviors between the participants with and without overweight/obesity status. Significant differences were found in the PE subscale of parenting practices, as well as lower fruit, and higher fried food, consumption.

Results of the univariate analysis on the associations of the background characteristics with unhealthy behaviors are listed in Table 3. Female children were less likely to have higher snack consumption (COR: 0.54, 95% CI: 0.32–0.91, *p* < 0.05) but were more likely to skip breakfast (COR: 1.70, 95% CI: 1.04–2.76, *p* < 0.05). Adolescent children were less likely to have insufficient fruit (COR: 0.55, 95% CI: 0.35–0.85, *p* < 0.01) and higher snack consumption (COR: 0.51, 95% CI: 0.32–0.81, *p* < 0.01). In addition, children with overweight and obese status were more likely to have higher fried food consumption (COR: 1.98, 95% CI: 1.22–3.21, *p* < 0.01) but less likely to have insufficient fruit consumption (COR: 0.56, 95% CI: 0.36–0.88, *p* < 0.05). Children with moderate ID (compared to the mild level), those with autism, and those with ADHD are at an elevated risk for some unhealthy eating behaviors, while those with Down syndrome and epilepsy were at reduced risk for consuming less vegetables, more fried food, and more sweetened drinks. In terms of parental characteristics, significantly reduced risks for higher snack consumption were observed among parent respondents at older ages (compared to those aged < 40 years), and parents with higher education levels (compared to those with junior secondary and below) were also associated with lower risks for lower vegetable consumption and breakfast skipping, while other paternal occupations (compared to administrators and professionals), divorced/separated/widowed parents (compared to married/cohabiting), and maternal obesity (compared to non-obesity) were significantly associated with increased risks of some child unhealthy behavior variables.

Table 4 presents the results of the multivariate logistic regression on associations of child body weight status and parenting practices with child unhealthy behaviors. In terms of main effects, children with overweight and obese status were more likely to engage in four out of seven unhealthy behaviors (including more sedentary behaviors, and higher fried food, higher sweetened drink, and higher snack consumption; AORs ranged from 1.51 to 1.96, *p* < 0.05), while they were at reduced risk for lower fruit consumption (AOR: 0.65, 95% CI: 0.43–0.99, *p* < 0.05). The RA subscale of parenting practices reduced the risks for higher fried food and sweetened drink consumption (AORs: 0.63 and 0.64, *p* < 0.05), while the UR subscale elevated the risk for higher fried food, higher sweetened drinks, and higher snack consumption, as well as breakfast skipping (AORs ranged from 1.28 to 1.60, *p* < 0.05). There were five interactions reaching significance, indicating that child body weight status moderated the associations between parenting practices and child behaviors, including RA with fried food, sweetened drinks, and breakfast skipping, and UR with vegetable intake and fried food (*p* < 0.05). The multivariate regression model for insufficient PA failed to build, as very few participants were available with sufficient MVPA (*n* = 34, Table 2).

Table 5 further presents results of those multivariate models involving significant interactions after stratification by child body weight status. Among children with non-overweight and obese status, parenting practices were not significantly associated with any child behavior except lower vegetable consumption (AOR: 0.68, 95%CI: 0.44–0.98, *p* = 0.040). For those with overweight and obese status, the RA subscale significantly reduced risks for children’s higher fried food and higher sweetened drinks consumption, and breakfast skipping (AORs ranged from 0.17 to 0.28, *p* < 0.01), whilst the UR practices elevated the risk for higher fried food consumption (AOR: 2.50, 95% CI: 1.36–4.60, *p* < 0.05). These associations were not significant among children with non-overweight and obese status.

## 4. Discussion

The present study was conducted to investigate the associations between parenting practices and child health-related behaviors, including an examination of the moderating role of child body weight status in their associations in a sample of children with ID in Hong Kong. Findings suggest that children of parents who used the RA subscale of parenting practices were less likely to develop unhealthy eating behaviors and children were more likely to access to unhealthy food if their parents used UR subscale, compared with children of parents who less frequently used these parenting practices. In addition, results indicated that child body weight status moderated the associations between UR and RA subscales and four out of seven behaviors (i.e., lower vegetable, higher fried food, and higher sweetened drink consumptions, as well as breakfast skipping). Significant associations were observed in children with overweight and obese status after stratifying for body weight status, except that UR was found to lower the risk of insufficient vegetable intake in children with non-overweight and obese status.

Our study found that the mean score of each subscale of parenting practices was similar between children with non-overweight and obese status and those without, except for the PE subscale, where parents of children with non-overweight and obese status showed higher scores. This indicates that parents were more likely to encourage their children to eat more, which is comparable with previous findings in typically developing children [45,46,47]. It is possible that parents of children with non-overweight and obese status have better knowledge of health and diet. As a result, they encourage children to consume more healthy food (e.g., vegetables), or eat food to maintain good weight status as a sign of health [48,49,50]. Future research is needed to better understand the parental motivation of using the PE subscale, especially in children with ID.

The close relationships between these obesogenic eating and activity behaviors and risk of overweight and obese status in children with and without ID have been well-documented in previous research [6,51,52]. Regarding the main effects of parenting practices, parents who used the RA subscale had children who consumed less fried food and less sweetened drinks; however, the use of UR made children more likely to consume fried food, sweetened drinks and snacks, and skip breakfast. Possible explanations may be that RA as a common adopted practice seems to be effective in reducing the opportunities to access unhealthy food. However, accumulating evidence showed that UR might promote overeating and interfere with children’s ability to self-regulate food consumption, leading to detrimental effects on eating behaviors [45]. There is little evidence examining these relationships in children with ID. In their counterparts, however, studies suggested conflicting results, with some studies confirming our results and some contradicting them [23,30,53]. There are several explanations for these findings. One explanation may be that previous studies adopted a large variation of items and subscales to measure parenting practices, which may not be comparable with other studies. For example, a study by Arredondo et al. found that the use of control subscales by parents, combining both techniques of UR and PE subscales, was associated with undesirable eating behaviors [23]. Moreover, a study by Gubbels et al. found that parental restriction, which contained items of both RA and UR subscales used in our study, was not associated with any of the eating behaviors [30]. Thus, it is difficult to compare the isolated effect of specific items or subscales in those findings with the findings of our study. Further research is needed to confirm the effects of RA and UR practices. Interaction between children and parents might be another explanation, indicating that the effects of parenting practices on child behaviors depended on the characteristics of children (e.g., eating style) and parents (e.g., education level), which might further influence the associations observed [30]. For example, previous evidence has demonstrated that associations between restriction and undesirable eating behaviors were observed only in children with deviant eating styles, and monitoring was associated with desirable diet in children, except those with deviant eating styles [30,31]. 

In addition, no significant associations were observed regarding MO, PE, and RF subscales in children with ID. Previous studies have reported conflicting results in these three subscales in typically developing children (MO (e.g., [15,23,30,54]); PE (e.g., [22,30]); RF (e.g., [23,55])). For instance, Arredondo et al. identified that parental monitoring and reinforcement may have a favorable influence on children’s eating behaviors [23]. This might be explained by the fact that typically developing children may have better awareness and responsiveness compared with children with ID when parents monitor their behaviors (MO) and praise them for healthy behaviors (RF). Moreover, no conclusive results of the effects of PE were observed. A previous study reported that parents who used higher levels of PE subscale had detrimental effects on children’s eating behaviors (i.e., lower fruits and vegetables consumption, higher high-fat foods consumption) [23,56], while another study suggested that children who exposed higher levels of PE were less likely to consume high-fat foods [57]. Except for eating behaviors, our study found no associations between each subscale of parenting practices and sedentary behaviors. As too few cases were grouped into sufficient MVPA (6.2%), we failed to examine the effects of parenting practices on insufficient MVPA. Previous studies showed mixed results in typically developing children. In line with our study, no associations between parenting practices and children’s PA and sedentary behaviors were observed by Arredondo et al. [23]. In contrast, a cohort study illustrated that the restriction of sedentary behaviors by parents was related to increased sedentary behaviors and decreased PA [30]. Therefore, future research should put more effort on examining the effects of activity-related parenting practices to yield firm conclusions.

Findings indicated that child body weight status significantly moderated the associations between parenting practices and several unhealthy child behaviors. Parents using RA would reduce the risk of developing several unhealthy eating behaviors in children with overweight and obese status, but not in children with non-overweight and obese status. In addition, children with overweight and obese status were more likely to consume more fried food, whereas children with non-overweight and obese status were less likely to intake insufficient vegetables if parents were adopting UR. There are no conclusive results from previous studies. Associations between parental restriction and desirable eating behaviors were stronger in children with higher body weight, in agreement with previous studies [30,58]. By contrast, another study demonstrated that child body weight status did not moderate the associations [23]. Regarding the observed favorable effect of UR on insufficient vegetable intake among children with non-overweight and obese status, it is difficult to explain why UR played a protective role in this association based on the existing literature. Therefore, more studies are needed to confirm these associations.

Several limitations should be noted in the current study. First, this study provided hints of associations, but could not infer causation due to the nature of cross-sectional study design. Thus, whether the studied children’s behaviors are the consequences of specific parenting practices or vice versa could not be established. In addition, characteristics of children and parents, children’s behaviors, and parenting practices were self-reported, which may cause recall bias and reporting bias. Therefore, objectively measurements (e.g., wearing an Actigraph to measure PA and sedentary behaviors) are suggested in future studies. Third, parenting practice measures have varied widely across studies, making comparisons between previous studies and our study more difficult. There is no consensus about how to measure parenting practices in an appropriate way. Future research should be directed at the development and testing of comprehensive and valid parenting practices measures [14]. Finally, although the study sample of children with ID in Hong Kong may limit the generalizability to broader population of children, this special pediatric population who need more attention is understudied, and findings will add much-needed evidence to the limited knowledge base in this population.

Considering that children with ID are more vulnerable to unhealthy behaviors, future research on exploring effective parenting practices to promote healthy behaviors is urgently needed, which calls for more longitudinal studies to confirm causal inferences. If replicated and confirmed, findings from this study may add an important evidence base to the development of educational interventions and help understand the extent to which parenting practices can be modified through intervention. This will afford clinicians the opportunity to advise parents on how to monitor and reinforce child health-related behaviors [14,23,59].

## 5. Conclusions

The current study found that child body weight status moderated the relationships between RA and UR parenting practices and some undesirable eating behaviors, but not sedentary behaviors. Significant effects of parenting practices were mostly observed in children with overweight and obese status, among whom appropriate restriction seems to have favorable effects on several eating behaviors, whereas the impact of UR practice depends on child body weight status. Overall, effective parenting practices worked particularly well for children with overweight and obese status, suggesting that parents should be encouraged to use RA and discouraged to use UR in children with overweight and obese status. Further research is needed to identify the effectiveness of parenting practices aimed at promoting healthy behaviors among children with and without ID. Then, research-based suggestions can be provided to parents regarding tailored practice for each individual child.

## Figures and Tables

**Table 1:** Background characteristics of the participants (*n* = 440).

	*n*	%
**Children’s characteristics**		
Gender		
Male	307	69.8
Female	133	30.2
Age group		
6–12 years	241	54.8
13–21 years	199	45.2
ID level		
Mild (IQ: 55–69)	309	72.4
Moderate (IQ: 35–54)	118	27.6
Autism		
No	170	38.9
Yes	267	61.1
ADHD		
No	289	66.1
Yes	148	33.9
Down Syndrome		
No	403	92.2
Yes	34	7.8
Epilepsy		
No	401	91.8
Yes	36	8.2
Body weight status ^a^		
Non-overweight/obese	301	68.4
Overweight/obese	139	31.6
**Respondents’ characteristics**		
Relationship with the children		
Mothers	348	79.1
Fathers	92	20.9
Age groups of the respondents		
<40 years	94	21.8
40–49 years	237	54.9
≥50 years	101	23.4
**Parental characteristics**		
Paternal education		
Junior secondary and below	128	29.7
Senior secondary	161	37.4
College or above	142	32.9
Maternal education		
Junior secondary and below	125	29.0
Senior secondary	188	43.6
College or above	118	27.4
Paternal occupation		
Administrators and Professionals	181	42.8
Others	242	57.2
Maternal occupation		
Housewives	214	49.9
Administrators and Professionals	91	21.2
Others	124	28.9
Parental marital status		
Married/cohabiting	376	86.2
Divorced/separated/widowed	60	13.8
Paternal obesity ^b^		
No	186	42.3
Yes	200	45.5
Missing	54	12.3
Maternal obesity ^b^		
No	297	67.5
Yes	119	27.0

Abbreviations: ID, intellectual disability; IQ, intelligence quotient; ADHD, attention deficit hyperactivity disorder; BMI, body weight index. Missing data < 6% were not presented in this table, which were also not counted when calculating percentages [44]. ^a^: Child overweight and obese status was identified using international age- and gender-specific criteria on BMI cut offs recommended by Cole [42]. ^b^: Parental obesity was defined using the BMI cutoff point of 25 kg/m^2^ for Asian adults [37].

**Table 2:** Distribution of scores of parenting practices (mean ± SD) and unhealthy behaviors (*n* and %) by child body weight status.

	All (*n* = 440)	Child Body Weight Status		*p*-Value
		Non-Overweight/Obese (*n* = 301)	Overweight/Obese (*n* = 139)	
	mean ± SD	mean ± SD	mean ± SD	*p*-value ^a^
**Subscale of parenting practices** (averaged score range: 1–5)				
Diet and PA monitoring (MO)	3.73 ± 0.65	3.73 ± 0.63	3.73 ± 0.70	0.987
Restricting access to unhealthy food and sedentary behaviors (RA)	3.68 ± 0.80	3.70 ± 0.80	3.64 ± 0.81	0.474
Pressure to eat more (PE)	3.05 ± 0.65	3.18 ± 0.63	2.75 ± 0.61	<0.001 ***
Reinforcement (RF)	4.25 ± 0.58	4.24 ± 0.59	4.27 ±0.57	0.669
Use food or sedentary behaviors as rewards (UR)	3.13 ± 0.81	3.10 ± 0.85	3.18 ± 0.74	0.368
	*n (%)*	*n (%)*	*n (%)*	*p*-value ^b^
**Unhealthy behaviors**				
Insufficient MVPA (<60 min/day)	406 (93.8)	280 (94.3)	126 (92.6)	0.525
More sedentary behaviors (≥4 h/day)	196 (47.7)	126 (44.5)	70 (54.7)	**0.070 ^†^**
Lower fruit consumption (<2 servings/day)	331 (75.7)	237 (79.3)	94 (68.1)	0.016 *
Lower vegetable consumption (<3 servings/day)	378 (86.5)	261 (87.3)	117 (84.8)	0.547
Higher fried food consumption (≥once/day)	86 (19.7)	48 (16.1)	38 (27.5)	0.007 **
Higher sweetened drink consumption (≥once/day)	200 (45.7)	128 (42.7)	72 (52.2)	**0.079 ^†^**
Higher snack consumption (≥twice/day)	104 (23.9)	66 (22.1)	38 (27.5)	0.229
Breakfast skipping (≤6 times/week)	89 (20.4)	62 (20.8)	27 (19.6)	0.800

Abbreviations: MVPA, moderate-to-vigorous intensity physical activity; SD, standard deviation. *p* values < 0.10 were bold. ^†^: *p* < 0.10; *: *p* < 0.05; **: *p* < 0.01; ***: *p* < 0.001. ^a^: Independent-samples t-tests were used to examine the differences in all subscales of parenting practices between children with non-overweight and obese status and those without. ^b^: Chi-square tests were used to examine the differences in unhealthy behaviors between children with non-overweight and obese status and those without.

**Table 3:** Associations between background characteristics and children’s unhealthy behaviors (CORs, 95% CIs).

	Insufficient MVPA (<60 min/day)	More Sedentary Behaviors (≥4 h/day)	Lower Fruit Consumption (<2 Servings/day)	Lower Vegetable Consumption (<3 Servings/day)	Higher Fried Food Consumption (≥Once/day)	Higher Sweetened Drink Consumption (≥Once/day)	Higher Snack Consumption (≥Twice/day)	Breakfast Skipping (≤6 Times/week)
**Children’s characteristics**								
Gender								
Male	1.00	1.00	1.00	1.00	1.00	1.00	1.00	1.00
Female	0.99 (0.42, 2.33)	**1.44 (0.94, 2.21) ^†^**	1.28 (0.78, 2.08)	1.19 (0.64, 2.20)	0.76 (0.46, 1.30)	0.83 (0.55, 1.25)	0.54 (0.32, 0.91) *	1.70 (1.04, 2.76) *
Age group								
6–12 years	1.00	1.00	1.00	1.00	1.00	1.00	1.00	1.00
13–21 years	0.64 (0.29, 1.41)	1.16 (0.79, 1.72)	0.55 (0.35, 0.85) **	**0.61 (0.35, 1.06) ^†^**	1.13 (0.71, 1.82)	1.00 (0.69, 1.46)	0.51 (0.32, 0.81) **	1.33 (0.83, 2.12)
Body weight status ^a^								
Non-overweight	1.00	1.00	1.00	1.00	1.00	1.00	1.00	1.00
Overweight/obese	0.77 (0.34, 1.72)	**1.50 (0.99, 2.29) ^†^**	0.56 (0.36, 0.88) *	0.81 (0.46, 1.44)	1.98 (1.22, 3.21) **	**1.47 (0.98, 2.20) ^†^**	1.34 (0.84, 2.12)	0.93 (0.56, 1.53)
ID level								
Mild (IQ: 55–69)	1.00	1.00	1.00	1.00	1.00	1.00	1.00	1.00
Moderate (IQ: 35–54)	**3.16 (0.93, 10.73) ^†^**	0.91 (0.59, 1.42)	**0.67 (0.41, 1.08) ^†^**	**0.61 (0.34, 1.09) ^†^**	1.16 (0.68, 1.95)	1.07 (0.70, 1.65)	1.81 (1.12, 2.93) *	0.96 (0.56, 1.63)
Autism								
No	1.00	1.00	1.00	1.00	1.00	1.00	1.00	1.00
Yes	**0.44 (0.18, 1.12) ^†^**	0.73 (0.49, 1.08)	0.74 (0.47, 1.18)	0.86 (0.49, 1.53)	1.38 (0.84, 2.28)	1.69 (1.14, 2.50) **	2.10 (1.29, 3.43) **	0.78 (0.49, 1.26)
ADHD								
No	1.00	1.00	1.00	1.00	1.00	1.00	1.00	1.00
Yes	1.24 (0.53, 2.90)	0.85 (0.56, 1.28)	0.61 (0.39, 0.95) *	0.78 (0.44, 1.37)	1.37 (0.84, 2.23)	1.13 (0.76, 1.68)	1.64 (1.04, 2.59) *	0.88 (0.53, 1.45)
Down Syndrome								
No	1.00	1.00	1.00	1.00	1.00	1.00	1.00	1.00
Yes	2.32 (0.31, 17.64)	1.62 (0.79, 3.29)	1.93 (0.73, 5.11)	0.90 (0.34, 2.43)	0.11 (0.02, 0.83) *	0.18 (0.07, 0.48) **	--	0.83 (0.33, 2.07)
Epilepsy								
No	1.00	1.00	1.00	1.00	1.00	1.00	1.00	1.00
Yes	--	0.69 (0.33, 1.42)	0.61 (0.29, 1.26)	0.43 (0.19, 0.97) *	1.62 (0.75, 3.51)	0.83 (0.41, 1.65)	0.62 (0.25, 1.53)	0.77 (0.31, 1.91)
**Parental characteristics**								
Respondents’ relationship with the children								
Mothers	1.00	1.00	1.00	1.00	1.00	1.00	1.00	1.00
Fathers	0.90 (0.35, 2.30)	0.94 (0.58, 1.52)	1.62 (0.90,2.93)	1.52 (0.72, 3.21)	1.40 (0.81, 2.43)	1.00 (0.63, 1.59)	1.00 (0.59, 1.72)	1.54 (0.90, 2.64)
Age group of the respondents								
<40 years	1.00	1.00	1.00	1.00	1.00	1.00	1.00	1.00
40–49 years	0.65 (0.21, 2.02)	0.73 (0.44, 1.20)	0.92 (0.51, 1.65)	0.85 (0.40, 1.82)	1.20 (0.64, 2.25)	1.16 (0.72, 1.88)	0.57 (0.34, 0.97) *	1.30 (0.71, 2.39)
≥50 years	0.49 (0.14, 1.70)	0.97 (0.54, 1.75)	**0.54 (0.28, 1.05) ^†^**	0.57 (0.25, 1.31)	1.28 (0.62, 2.63)	0.67 (0.38, 1.19)	0.33 (0.17, 0.67) **	0.98 (0.47, 2.04)
Paternal education								
Junior secondary and below	1.00	1.00	1.00	1.00	1.00	1.00	1.00	1.00
Senior secondary	1.48 (0.58, 3.75)	0.74 (0.46, 1.21)	1.01 (0.59, 1.74)	0.74 (0.35, 1.57)	1.01 (0.57, 1.79)	1.03 (0.64, 1.64)	1.38 (0.78, 2.42)	0.54 (0.31, 0.93) *
College or above	1.48 (0.57, 3.88)	1.06 (0.65, 1.74)	1.04 (0.59, 1.81)	0.45 (0.22, 0.93) *	0.77 (0.41, 1.42)	0.72 (0.44, 1.16)	1.36 (0.76, 2.42)	0.31 (0.16, 0.58) ^***^
Maternal education								
Junior secondary and below	1.00	1.00	1.00	1.00	1.00	1.00	1.00	1.00
Senior secondary	0.70 (0.26, 1.90)	1.06 (0.66, 1.71)	0.92 (0.53, 1.59)	0.67 (0.32, 1.43)	1.04 (0.59, 1.82)	1.15 (0.73, 1.80)	1.42 (0.80, 2.51)	0.46 (0.27, 0.79) **
College or above	0.72 (0.24, 2.15)	0.91 (0.54, 1.53)	0.70 (0.39, 1.25)	0.43 (0.20, 0.93) *	0.74 (0.38, 1.43)	**0.64 (0.38, 1.08) ^†^**	**1.82 (0.99, 3.35) ^†^**	0.28 (0.12, 0.48) ***
Paternal occupation								
Administrators and Professionals	1.00	1.00	1.00	1.00	1.00	1.00	1.00	1.00
Others	0.77 (0.34, 1.72)	0.82 (0.55, 1.22)	0.75 (0.48, 1.18)	1.27 (0.72, 2.23)	1.32 (0.80, 2.17)	1.48 (1.00, 2.19) *	0.71 (0.45, 1.11)	2.01 (1.20, 3.37) **
Maternal occupation								
Housewives	1.00	1.00	1.00	1.00	1.00	1.00	1.00	1.00
Administrators and Professionals	**0.41 (0.16, 1.02) ^†^**	0.69 (0.42, 1.15)	0.79 (0.46, 1.38)	0.71 (0.37, 1.39)	1.00 (0.53, 1.91)	1.03 (0.63, 1.69)	1.29 (0.73, 2.27)	**0.49 (0.24, 1.00) ^†^**
Others	0.84 (0.31, 2.28)	**0.64 (0.40, 1.01) ^†^**	1.14 (0.67, 1.93)	1.20 (0.60, 2.37)	**1.59 (0.92, 2.72) ^†^**	1.30 (0.83, 2.02)	1.16 (0.68, 1.96)	1.06 (0.62, 1.80)
Parental marital status								
Married/cohabiting	1.00	1.00	1.00	1.00	1.00	1.00	1.00	1.00
Divorced/separated/widowed	0.93 (0.31, 2.78)	1.19 (0.67, 2.12)	1.69 (0.82, 3.45)	3.36 (1.02, 11.09) *	1.48 (0.78, 2.80)	0.83 (0.48, 1.44)	0.88 (0.45, 1.69)	1.39 (0.74, 2.64)
Paternal obesity ^b^								
No	1.00	1.00	1.00	1.00	1.00	1.00	1.00	1.00
Yes	0.78 (0.35, 1.74)	0.96 (0.63, 1.44)	1.18 (0.74, 1.88)	0.74 (0.41, 1.33)	0.71 (0.43, 1.19)	0.77 (0.52, 1.15)	0.80 (0.50, 1.27)	0.77 (0.46, 1.28)
Missing	3.18 (0.40, 25.22)	1.08 (0.56, 2.10)	1.21 (0.59, 2.48)	1.06 (0.41, 2.78)	1.20 (0.59, 2.46)	0.76 (0.41, 1.40)	**0.46 (0.20, 1.05) ^†^**	1.61 (0.81, 3.19)
Maternal obesity ^b^								
No	1.00	1.00	1.00	1.00	1.00	1.00	1.00	1.00
Yes	0.60 (0.26, 1.36)	1.11 (0.71, 1.72)	0.89 (0.55, 1.47)	0.78 (0.43, 1.44)	2.32 (1.40, 3.85) **	1.28 (0.83, 1.97)	1.19 (0.73, 1.93)	1.41 (0.84, 2.37)

Abbreviations: CORs, crude odds ratios; 95% CIs, 95% confidence intervals; MVPA, moderate-to-vigorous intensity physical activity; ID, intellectual disability; IQ, intelligence quotient; ADHD, attention deficit hyperactivity disorder; BMI, body weight index. Data are presented with CORs and their 95% CIs are derived from univariate logistical regression. Those with *p* < 0.10 were bold, which would be adjusted in further regressions. ^†^: *p* < 0.10; *: *p* < 0.05; **: *p* < 0.01; ***: *p* < 0.001. “--” indicates that the counts in some groups were zero and CORs were not calculated. ^a^: A child’s overweight and obese status was identified using international age- and gender-specific criteria on BMI cut offs recommended by Cole [42]. ^b^: Parental obesity was defined using the BMI cutoff point of 25 kg/m^2^ for Asian adults [37].

**Table 4:** Multivariate logistic regression on associations of child body weight status and parenting practices with child unhealthy behaviors (AORs, 95% CIs).

	More Sedentary Behaviors (≥4 h/day)	Lower Fruit Consumption (<2 Servings/day)	Lower Vegetable Consumption (<3 Servings/day)	Higher Fried Food Consumption (≥Once/day)	Higher Sweetened Drink Consumption (≥Once/day)	Higher Snack Consumption (≥Twice/day)	Breakfast Skipping (≤6 Times/week)
**Main effects**							
*Body weight status of the children*							
Non-overweight/obese	1.00	1.00	1.00	1.00	1.00	1.00	1.00
Overweight/obese	1.51 (1.02, 2.24) *	0.65 (0.43, 0.99) *	0.81 (0.46, 1.42)	1.96 (1.24, 3.12) **	1.73 (1.16, 2.57) **	1.56 (1.01, 2.41) *	0.86 (0.52, 1.42)
*Parenting practices*							
MO	0.80 (0.50, 1.28)	0.62 (0.37, 1.05)	0.58 (0.27, 1.24)	0.59 (0.33, 1.5)	0.64 (0.39, 1.04)	0.73 (0.43, 1.24)	0.58 (0.33, 1.03)
RA	1.38 (0.93, 2.04)	1.16 (0.76, 1.78)	0.71 (0.38, 1.33)	0.63 (0.40, 1.00) *	0.64 (0.43, 0.96) *	0.90 (0.59, 1.38)	0.69 (0.43, 1.01)
PE	1.01 (0.75, 1.37)	1.04 (0.74, 1.45)	1.19 (0.75, 1.88)	1.05 (0.71, 1.56)	1.03 (0.75, 1.40)	1.16 (0.82, 1.64)	1.14 (0.77, 1.70)
RF	0.86 (0.63, 1.17)	0.99 (0.70, 1.41)	0.96 (0.58,1.57)	0.83 (0.56, 1.22)	0.88 (0.63, 1.22)	0.96 (0.68, 1.37)	0.96 (0.66, 1.39)
UR	0.95 (0.76, 1.20)	0.90 (0.70, 1.17)	0.86 (0.61, 1.21)	1.43 (1.04, 1.95) *	1.51 (1.18, 1.94) **	1.60 (1.21, 2.11) **	1.28 (0.93, 1.75) *
**Interactions**							
Body weight status * RA	--	--	--	0.33 (0.12, 0.96) *	0.32 (0.12, 0.88) *	--	0.19 (0.06, 0.63) *
Body weight status * UR	--	--	2.34 (1.10, 5.00) *	2.33 (1.07, 4.20) *	--	--	--

Abbreviations: AORs, adjusted odds ratios; 95% CIs, confidence intervals; MO, diet and physical activity monitoring; RA, restricting access to unhealthy food and sedentary behaviors; PE, pressure to eat more; RF, reinforcement; UR, use food or sedentary behaviors as rewards. Data are presented with AORs and their 95% CIs derived from multivariate logistic regression, where child body weight status and the five subscales of parenting practices were forcedly entered in Block 1, along with children’s gender, age, and those background characteristics with *p* < 0.10 in univariate analysis (as shown in Table 3); interaction items between child body weight status and each subscale of parenting practices were forward selected in Block 2, with *p* = 0.10 and *p* = 0.15 as entry and removal criteria, respectively. Only interactions with *p* < 0.05 were presented in this table. AORs with *p* < 0.05 were bold. *: *p* < 0.05; **: *p* < 0.01.

**Table 5:** Associations between parenting practices and children’s unhealthy behaviors after stratification by child body weight status.

	Non-Overweight/Obese Children		Overweight/Obese Children	
	AOR (95% CI)	*p* Value	AOR (95% CI)	*p* Value
Lower vegetable consumption (<3 servings/day)				
UR	0.68 (0.44, 0.98)	0.040 *	1.58 (0.83, 3.02)	0.170
Higher fried food consumption (≥ once/day)				
RA	0.89 (0.51, 1.57)	0.700	0.28 (0.11, 0.71)	0.007 **
UR	1.15 (0.79, 1.68)	0.460	2.50 (1.36, 4.60)	0.003 **
Higher sweetened drink consumption (≥ once/day)				
RA	0.83 (0.52, 1.31)	0.410	0.24 (0.09, 0.64)	0.004 **
Breakfast skipping (≤6 times/week)				
RA	1.01 (0.59, 1.75)	0.960	0.17 (0.06, 0.55)	0.003 **

Abbreviations: AOR, adjusted odds ratio; 95% CI, confidence interval; UR, use food or sedentary behaviors as rewards; RA, restricting access to unhealthy food and sedentary behaviors. AORs and 95%CIs were derived from multivariate logistical regression for children with and without overweight/obese status separately, variables adjusted were same as in Table 4. Significant *p* values were bold. *: *p* < 0.05; **: *p* < 0.01.

## Data Availability

The data that support the findings of this study are available on request from the corresponding author [Y.G.]. The data are not publicly available due to restrictions (e.g., containing information that could compromise the privacy of research participants).

## Supplementary Materials

The following supporting information can be downloaded at: https://www.mdpi.com/article/10.3390/nu14245206/s1. Table S1: Items and subscales of the scale of parenting practices.
