# Supplementary material for: The Relationships between Parenting Practices and Child Health-Related Behaviors in Children with Intellectual Disability: The Moderating Role of Child Body Weight Status
Source: Nutrients. 2022 Dec 7;14(24):5206. doi: 10.3390/nu14245206 (PMC9784023; doi:10.3390/nu14245206)
Supplement: Supplementary file 1 [file nutrients-14-05206-s001.zip › nutrients-2050461-supplementary.pdf]

Table S1 Items and subscales of the scale of parenting practices *

Item	Rating
**Diet and PA monitoring (MO, 6 items)**	
How much do you keep track of sweet snacks (such as cake, candy, ice cream, etc.) that your child eats?	From 1=never to 5=always
How much do you keep track of high-fat foods (such as fried food, puffed food, etc.) that your child eats?	From 1=never to 5=always
How much do you keep track of servings of fruit & vegetable that your child eats?	From 1=never to 5=always
My child has to get my permission before eating snacks.	From 1=never to 5=always
How much do you keep track of the amount of TV or videos your child is watching?	From 1=never to 5=always
How much do you keep track of the intensity and duration of PA of your child?	From 1=never to 5=always
**Restricting access to unhealthy food and sedentary behaviours (RA, 4 items)**	
I limit the amount of sweetened drinks (e.g. coke, sprite, orange juice) my child drinks.	From 1=never to 5=always
I limit the number of snacks my child eats.	From 1=never to 5=always
On weekdays, I limit the amount of TV or videos my child is watching.	From 1=never to 5=always
On weekends, I limit the amount of TV or videos my child is watching.	From 1=never to 5=always
**Pressure to eat more (PE, 3 items)**	
I have to be especially careful to make sure my child eats enough.	From 1=strongly disagree to 5=strongly agree
I let my child eat more even if he/she says "I am full".	From 1=strongly disagree to 5=strongly agree
My child eats less than what he/she should eat if I do not intervene.	From 1=strongly disagree to 5=strongly agree
**Reinforcement regarding children's eating and PA (RF, 2 items)**	
I praise my child if he/she eats healthy food.	From 1=strongly disagree to 5=strongly agree
I praise my child if he/she actively increases PA.	From 1=strongly disagree to 5=strongly agree
**Using food or sedentary behaviours as rewards (UR, 2 items)**	
I would offer some snacks (such as candy, ice cream, etc.) as a reward for good behaviours.	From 1=strongly disagree to 5=strongly agree
I would offer TV, video, or video games to my child as a reward for good behaviours.	From 1=strongly disagree to 5=strongly agree

*: the scale was developed by Wen & Hui, 2012 for Chinese children and possesses sound validity and reliability.

Abbreviations: PA, physical activity.

父母教養行為問卷

請選擇與您想法和行為一致的表述，選項從左至右的行為頻率或同意程度依次增高，請選出最符合您情況的答案。

下述題目詢問您行為發生的頻率	從不	很少	有時	經常	總是
1. 我會關注我的孩子吃甜食的數量（如蛋糕、糖果、霜淇淋）	①	②	③	④	⑤
2. 我會關注我的孩子吃高油脂食品的數量（如：油炸、膨化食品）	①	②	③	④	⑤
3. 我會關注我的孩子吃蔬菜水果的數量	①	②	③	④	⑤
4. 我的孩子吃零食之前要經過我同意	①	②	③	④	⑤

5. 我會關注我的孩子看電視、玩電子遊戲、和上網的時間	①	②	③	④	⑤
6. 我會關注我的孩子體育鍛煉的運動強度和時間	①	②	③	④	⑤
7. 我會控制我的孩子喝含糖飲料的量（可樂、雪碧、鮮橙多等）	①	②	③	④	⑤
8. 我會控制我的孩子吃零食的數量	①	②	③	④	⑤
9. 週一到週五，我會控制我的孩子看電視、玩電子遊戲的時間。	①	②	③	④	⑤
10. 週末，我會控制我的孩子看電視、玩電子遊戲的時間。	①	②	③	④	⑤

下述題目詢問您對於事情的看法	完全不同意	不同意	中立	同意	完全同意
11. 我應該特別關注，確保讓我的孩子吃飽飯	①	②	③	④	⑤
12. 假如我的孩子說「我吃飽了」，我還是會儘量讓他/她再吃一些	①	②	③	④	⑤
13. 假如我不對孩子的飲食加以指導和調節，他會吃得比他該吃的少	①	②	③	④	⑤
14. 假如我的孩子選擇吃健康的食品，我會表揚他/她	①	②	③	④	⑤
15. 假如我的孩子積極參加體育運動，我會表揚他/她	①	②	③	④	⑤
16. 假如孩子表現好，我會給些零食（糖果、冰淇淋等）獎勵他/她	①	②	③	④	⑤
17. 假如孩子表現好，我會讓他/她看電視或者玩電子遊戲作為獎勵	①	②	③	④	⑤
